# Nonalcoholic fatty liver disease: epidemiology, pathogenesis and therapeutic implications

**Published:** 2018

**Authors:** Craita Isabela Andronescu, Monica Roxana Purcarea, Petru Aurel Babes

**Affiliations:** * Medical Center Medas, Bucharest, Romania; ** Clinical Nephrology Hospital “Carol Davila”, Bucharest, Romania; *** University of Oradea, Faculty of Medicine and Pharmacy, Oradea, Romania

**Keywords:** central obesity, Type 2 DM, insulin resistance, cryptogenic cirrhosis, hepatocellular carcinoma

## Abstract

The increase of the prevalence of nonalcoholic fatty liver disease in the context of the explosive epidemy of obesity worldwide over the last decades and the emergence of new effective therapies for viral hepatitis has brought this entity to the forefront of hepatologist concerns. Today is a certainty that fatty liver can complicate with cirrhosis and hepatocellular carcinoma; moreover, nonalcoholic fatty liver disease is the main cause of cryptogenic cirrhosis and the second cause of liver transplantation.

This review revises the epidemiology of the disease, brings forward some progress in pathogenesis and outlines the directions to be followed in nonalcoholic fatty liver disease prevention and therapy.

Today, nonalcoholic fatty liver disease is considered to be the liver manifestation of metabolic syndome, with its same prevalence (20-30%). If the patients do not die through cardiovascular disease, it can lead to serious liver complications.

## Introduction

Nonalcoholic fatty liver disease (NAFLD)is a common disease characterized by liver steatosis (accumulation of triglycerides in the liver) excluding other causes of secondary steatosis (alcohol consumption > 40 grams / week, hypothyroidism, glucocorticoids, tamoxiphene, etc).

It is known that 25% of adults with fatty liver have nonalcoholic steatohepatitis; we can think of this possibility when the serum levels of aminotransferases are increased in the absence of other liver lesions. An ALT / AST ratio in favor of ALT characterizes nonalcoholic steatohepatitis (NASH); an AST / ALT ratio in favor of AST is found in nonalcoholic steatohepatitis with cirrhosis.

Nonalcoholic fatty liver disease may be complicated by cirrhosis or hepatocellular carcinoma (HCC) and it is the most important cause of cryptogenic cirrhosis. This evolution is more common in those with nonalcoholic steatohepatitis in whom we are witnessing the disappearance of steatosis once the patient becomes catabolic by cirrhosis. The nonalcoholic fatty liver diseasespectrum comprises simple steatosis (without inflammation) and NASH (with necroinflammatory lesions) that can ultimately lead to fibrosis and cirrhosis. [**1**][**2**]

## Epidemiology

Nonalcoholic fatty liver disease is today the most common liver disease. It is estimated that in Europe, the average prevalence is 25-26% and in the USA about 25% of the adults have fatty liver. In Romania, 20% of adults have NAFLD. Studies in Romania used ultrasound diagnostic as in Italy, Spain, UK; in Italy and Spain there were slightly higher results than in our country (**Table 1**); in Germany, where fatty liver index (FLI) was used, the prevalence was 33% and in Greece 31% where histological evaluation was used. In the UK, the statistical outcomes reached 46%, but it was noted that the patients being examined were diabetics. [**3**]

**Table 1:** Prevalence of NAFLD in different population in Europe (after S. Bellentani)

	Case Identification	Prevalence NAFLD
14 Countries	FLI	33 % (adults)
Germany	US and LE	2% (36% in obese children)
Germany	US	30% (adults)
Greece	Histology	31% (adults)
Italy	US	26% (adults)
Italy	US	12.5% (adolescents)
Italy	US	44% (obese children)
Italy	US	69.5% (diabetic pts)
Romania	US	20% (adults)
Spain	US	25.8% (adults)
UK	US	46.2% (diabetic pts)
**FLI**, fatty liver index; **US**, ultrasound; **LE**, liver enzymes					

The disease reaches a peak in the 5th and 6th decades of life. Female sex is more affected (65% - 83% of cases). However, female sex is not a risk factor for nonalcoholic fatty liver disease. Estrogens play a protective role, proof that the risk of nonalcoholic steatohepatitis in women is increased especially after the menopause when the prevalence peaks (in the 5th and 6th decade) [1,2]. At the same time, NAFLD and NASH are increasingly noticed in children [6]. A Danish study of more 250 000 children pointed that obese children are predisposed to hepatocellular carcinoma, later in their adulthood.

## Major risk factors

It has been observed that in some populations, the nonalcoholic fatty liver disease risk increases significantly. There is a clear association between nonalcoholic fatty liver disease and other elements of the metabolic syndrome (MS): DZ, obesity, hyperlipidemia. Today, nonalcoholic fatty liver disease is considered to be the hepatic component of MS. All of these risk factors favornonalcoholic fatty liver disease by developing insulin resistance [**5**]. This results in increased hepatic accumulation of fatty acids.

• Obesity The nonalcoholic fatty liver disease prevalence is increased in overweight or obese people. Wanless and Lentz in an autopsy study found steatosis in 70% of the obese and 35% in the normoponderal individuals; accordingly, NASH was found in 18.5% of the obese versus 3.5% of the normoponderal individuals [**2**][**6**].

• DZT2 The prevalence of nonalcoholic fatty liver disease among patients with DZT2 is estimated at 75%. At the same time, patients with DZT2 have an increased risk of NASH and advanced fibrosis. Of the 42 patients followed by Powell & col, 15 (37%) had hyperglycemia [**7**]. In DZT1, NAFLD is rare, an additional argument for the role of obesity and insulin resistance in the pathogenesis of the disease.

• Hyperlipidemia Studies have revealed a vast range of results (20% - 81%) in terms of association between nonalcoholic fatty liver disease and cholesterol and serum triglyceride levels.

In the Powell study quoted above, 26 patients (61%) experienced hyperlipidemia. Hypertriglyceridemia à jeun, commonly seen in nonalcoholic fatty liver disease, is a physiological event, and cannot prove an etiological role in the production of this condition. [**7**]

The role of hypercholesterolemia in nonalcoholic fatty liver disease determination is not proven. The clear benefit of statin treatment in these patients is the reduction of cardiovascular risk without reducing the progression to fibrosis.

## Other risk factors

Rapid weight loss and jejuno-ileal bypass are other risk factors for nonalcoholic fatty liver disease. Approximately 2.2% - 6% of patients who rapidly lose weight have abnormalities in liver function during the first 18 months. In a study, Mc Farland & col. have demonstrated that 40% of patients with jejuno-ileal bypass have liver function disorder. [**8**]

The most common medicines that can produce nonalcoholic fatty liver disease are amiodarone, glucocorticosteroids, tetracyclines, zidovudine. Note that the infusions of i.v. glucose and total parenteral nutrition may be the cause of NAFLD. It should not be forgotten that sedentary behavior, sweet drinks increase insulin resistance. Based on the experimental model, the trans fat-containing diet produces NASH.

The true stake of NAFLD is the progression to advanced fibrosis [**9**]. It is considered advanced fibrosis F≥2 on a scale from F0 to F4 as follows:

• F0 (without fibrosis) • F1 (portal fibrosis without sepsis) • F2 (portal fibrosis with fewer sepsis) • F3 (bridging septa between the central veins and the portal veins) • F4 (cirrhosis)

The risk factors of NASH evolution to advanced fibrosis can be subdivided into:

• Local histological factors o Hepatic inflammation o Balloon degeneration plus Mallory bodies o Fibrosis

• Systemic factors o Advanced age o DZ o BMI ≥ 28 kg/m^2^ o A visceral adiposity index that includes waist circumference, BMI, serum levels of TG and HDL.

## Natural history

There are few prospective studies on nonalcoholic fatty liver disease patients. The evolution of the disease is not sufficiently well understood only on the basis of available retrospective studies. The survival estimated at 5 and 10 years of nonalcoholic steatohepatitis patients is 67% and 59% respectively; it is still difficult to distinguish the cause of death in these patients which may also be due to cardiovascular disease, complications of diabetes and other manifestations of the metabolic syndrome [**2**][**4**].

Analyzing the studies available, we can state that 23% of NASH patients will develop to fibrosis and 15% have a risk of developing cirrhosis in 5 years. Note that 12% register biological improvement by regressing to simple steatosis [**6**].

Powell & col. followed 42 patients for an average period of 4.5 years. Out of these, 18 patients had fibrosis, 2 patients developed severe fibrosis and 1 patient had cirrhosis at the initial biopsy exam. During the study, one patient progressed from fibrosis to cirrhosis, one patient died from hepatocellular carcinoma. The two patients with severe fibrosis developed cirrhosis [**2**][**7**].

Lee & col. tracked 39 NASH patients approximately 3.8 years. During this time, a patient died by liver decompensation, 5 out of 13 patients who underwent a new liver biopsy showed fibrosis progression and 2 of them evolved to cirrhosis [**10**].

On the other hand, Lindor & col. in 2004 on a randomized controlled trial using ursodeoxycholic acid for the treatment of NASH, noticed a significant improvement in steatosis and fibrosis even in the control group [**11**]. Also in 2004, Adams & col. on a longitudinal study, although reported an increased mortality compared to the general population, found that only 3% of those with NASH developed cirrhosis and only 1.7% died due to liver disease.

As a result, Adams & col concluded that increased overall mortality was caused by cardiovascular disease in MS patients [**2**][**12**].

In general, the progression to fibrosis is slow, at a stage per decade. This means that those with F2 will develop into cirrhosis in 20 years. This conclusion was based on studies conducted on biopsy specimens. But there is clinical practice and exceptions to this rule. There are situations when fibrosis diminishes rapidly with NASH improvement; sometimes fibrosis progresses despite the fact that NASH has resolved. There are likely rates of individual progression or regression of fibrosis in patients with poorly understood NASH. It is possible that the severity of the inflammation will determine and influence the evolution to cirrhosis (proven by biopsy)[**4**].

As 6% of the adult population in the US has NASH, and at least 25% have F2 at the time of diagnosis, the American authors predict that in the near future, NASH will be the first cause of transplant.

## Hepatocellular carcinoma

Welzel & col. (2013), Wong & col (2016) have conducted studies that have shown an increase in the incidence of primary hepatic cancer. The incidence of the disease is about 1-2% [**13**][**14**].

It should be noted that the cancer process is also found in the fatty liver without cirrhosis. For this reason, regular imaging screening is useful for MS patients.

## Pathogenesis and therapeutic implications

Nonalcoholic steatohepatitis is produced by the lipotoxic injury of the hepatocytes. It has been shown that triglycerides have no toxic effect on hepatocytes.

The cause of lipotoxicity seems to be represented by free fatty acids (FFA) metabolites still inadequately identified (possibly ceramides, diacylglycerols, omega-oxidized fatty acids, etc.).

Hepatocyte lesions produced in NASH are due to several mechanisms that are interested in the metabolism of fatty acids.

The primary metabolic defect refers to increased peripheral mobilization of fatty acid (F.A). Within this mechanism, insulin resistance plays a major role. Insulin normally prevents fatty tissue lipolysis. Insulin resistance and hyperinsulinemia cause lipolysis, increasing the level in the circulation of fatty acids [**1**][**2**][**15**].

Other incriminated mechanisms are:

• Increased hepatic synthesis of FA. This is made from carbohydrates presented in excess in the liver which are converted in FA through lipogenesis. Similarly, food carbohydrates (sweet drinks, parenteral nutrition with i.v. glucose) results in hepatic lipotoxicity. • Impaired of hepatic catabolism of FA. This can occur by disrupting the mitochondrial beta-oxidation of AG leading to NAFLD and NASH. Defects of mitochondrial function (alcohol, acute fatty liver of pregnancy) and other oxidative pathways (cytochrome P-450, omega-oxidation, etc.) may appear. • Dysregulated triglyceride synthesis and VLDL secretion by the liver. Nonmethabolized FA in the liver are converted into TG by re-esterification. For this process, monounsaturated fatty acids (MUFAs) are required. MUFA degraded synthesis produces lipotoxicity [**1**][**2**][**15**].

Dysregulation of the steps described above produces liver steatosis. At the hepatocyte level, accumulated triglycerides can release FFA through the autophagy mechanism [**1**].

The damaged hepatocytes release factors that lead to the accumulation of immune cells, miofibroblasts that through a vicious circle maintain and aggravate inflammation [**4**].

Affected hepatocytes trigger repair mechanisms for replacement with other hepatocytes. If the destructive factor that promotes inflammation persists (metabolic syndrome etc.) the regenerative process, the reparatory process is disrupted leading to fibrosis and cirrhosis and consequently to cancer risk.

The angular stone of any therapeutic plan should take into account weight loss and physical effort. Weight loss should be gradual (aprox 1.5 kg/week). Weight loss of 7% is optimal [**4**][**16**][**17**][**18**][**19**].

It should be noted that weight loss accompanied by protein malnutrition does not solve liver steatosis. Patients who lose weight through bariatric surgery procedures should be cautious after surgery to avoid nutritional deficiencies. Alcohol consumption should be forbidden in patients with fibrosis. Other patients without fibrosis can consume limited and occasionally alcohol. Caffeine coffee consumption (2 cups / day) reduces the risk of fibrosis in NAFLD.

There is no approved medication for NASH treatment.

Several drugs have been tested in clinical trials

• Pioglitazone increases insulin sensitivity and prevents lipolysis • Vitamin E • Other drugs are under study: PPAR α and PPAR δ agonists, obetricholic acid, chemokinetic 2 and 5 receptor antagonists, etc [**4**][**19**].

## Conclusions

Nonalcoholic steatohepatitis is “de facto” the hepatic component of MS. Unspecified 40 years ago, today it is considered the main cause of cryptogenic cirrhosis and it is a risk factor for primitive liver cancer even in the absence of cirrhosis. Pathogenically, it has as fundamental factors insulin resistance, hyperinsulinemia as well as the oxidative stress associated mainly with S.M. 

The essential treatment consists of weight loss and constant physical effort. Several drugs are tested in clinical trials.

## Conflict of interest

The authors declare no conflict of interest.
